# Unusual timing of a common complication after pulmonary vein isolation

**DOI:** 10.1007/s12471-017-1027-7

**Published:** 2017-08-01

**Authors:** K. Van Kolen, E. Ströker, Y. De Greef

**Affiliations:** Department of Cardiology – Electrophysiology Unit, ZNA Middelheim, Antwerp, Belgium

**Fig. 1:**
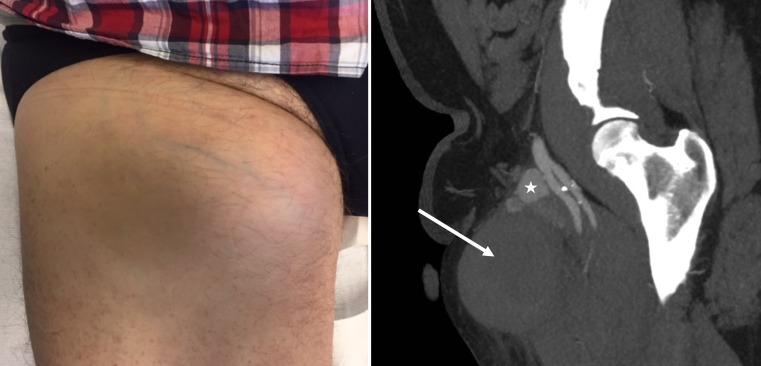
Clinical presentation of the large right groin mass (*left panel*). CT angiography demonstrating a pseudo-aneurysm of the femoral artery with an active component (*asterisk*), and a large haematoma anteriorly (*arrow*) (*right panel*)

A 57-year-old patient presents with a large mass at the right groin two months after cryoballoon-guided pulmonary vein isolation (PVI) (Fig. 1, *left panel*). A pseudo-aneurysm of the superficial femoral artery is seen on CT with a small, encapsulated active bleeding region (asterisk) surrounded by a large haematoma (arrow, Fig. 1, *right panel*).

The pseudo-aneurysm of our patient was clinically missed the day after the procedure. Progressive swelling occurred during the first days and weeks after PVI, however, since paucisymptomatic and not worried about his condition, he waited to seek medical advice until the planned follow-up consultation 2 months after PVI. The two-phase time-frame of a small initial pseudo-aneurysm with subsequent rupture of the wall creating a surrounding haematoma is the most probable explanation.

Vascular pseudo-aneurysms are reported in up to 2.4% of patients after PVI [1, 2]. The late presentation, large size and clinically silent course are unusual. Systematic use of ultrasound post-procedure might be useful to detect these ‘asymptomatic’ pseudo-aneurysms as early as possible. More importantly, this complication might be totally avoided by using ultrasound to guide vascular access during the procedure.
